# Supplementary material for: The immune response to a fungus in pancreatic cancer samples
Source: bioRxiv. 2023 Mar 29:2023.03.28.534606. Preprint. [Version 1] doi: 10.1101/2023.03.28.534606 (PMC10081247; doi:10.1101/2023.03.28.534606)
Supplement: Supplement 1 [file NIHPP2023.03.28.534606v1-supplement-1.pdf]

bioRxiv preprint doi: https://doi.org/10.1101/2023.03.28.534606; this version posted March 29, 2023. The copyright holder for this preprint (which was not certified by peer review) is the author/funder, who has granted bioRxiv a license to display the preprint in perpetuity. It is made available under aCC-BY 4.0 International license.

Supplemental Figures

Figure S1

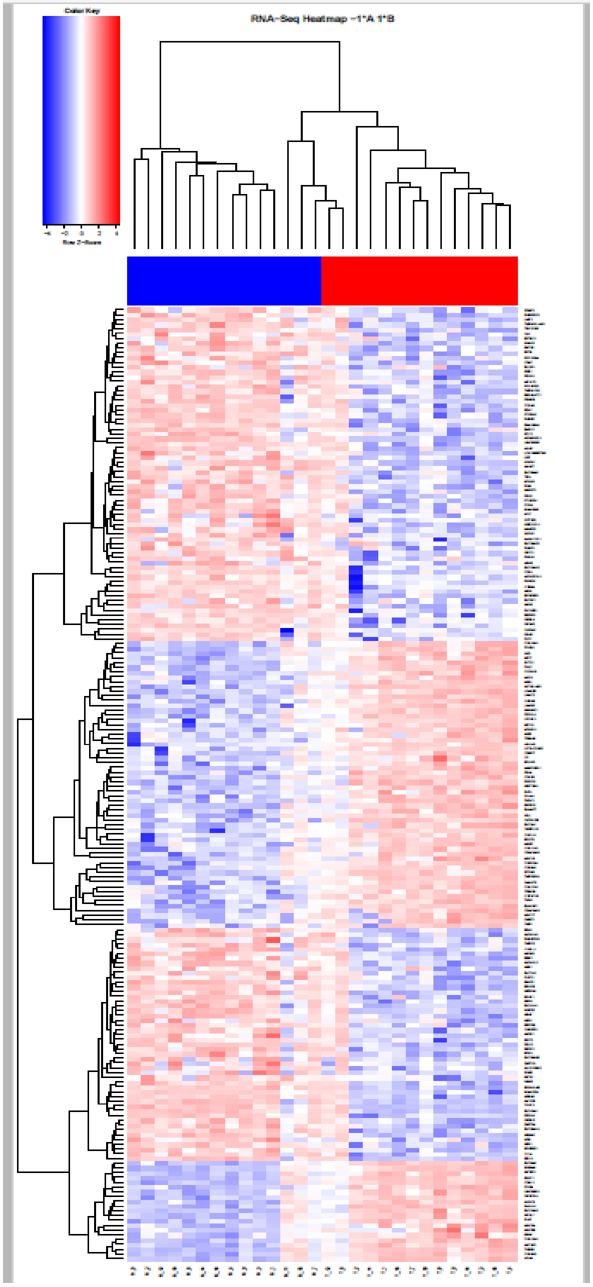

Figure S1: Heatmap summarizing the supervised clustering and differential gene expression analysis comparing the normal samples to the tumor samples. Sample names are along the bottom, gene names are along the right side of the figure.

bioRxiv preprint doi: https://doi.org/10.1101/2023.03.28.534606; this version posted March 29, 2023. The copyright holder for this preprint (which was not certified by peer review) is the author/funder, who has granted bioRxiv a license to display the preprint in perpetuity. It is made available under aCC-BY 4.0 International license.

Figure S2

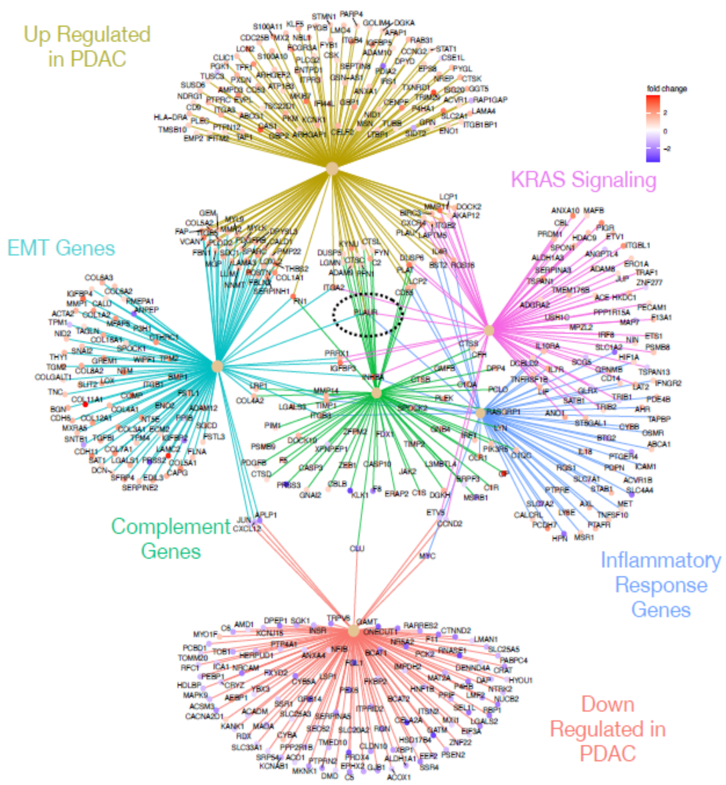

Figure S2: CNET plot showing gene overlaps in enriched genesets. Geneset enrichment analysis of genes differentially expressed between tumor and normal samples indicated enrichment for sets of genes involved in KRAS signaling (purple), inflammatory response (blue), complement cascade (green), and epithelial mesenchymal transition (turquoise). Figure also includes genes known to up (orange) or down (olive) regulated in PDAC. Black dashed circle indicates PLAUR, which connects to all 5 of the 6 genesets (not connected to down in PDAC). Genes at the end spokes indicate differential expressed in our dataset that were enriched in each geneset, color of dots indicated whether the gene was up (red tones) or down (blue tones) regulated in tumors relative to normal samples. (17, 21, 34).

bioRxiv preprint doi: https://doi.org/10.1101/2023.03.28.534606; this version posted March 29, 2023. The copyright holder for this preprint (which was not certified by peer review) is the author/funder, who has granted bioRxiv a license to display the preprint in perpetuity. It is made available under aCC-BY 4.0 International license.

Table S1: RNA sequencing statistics by sample.

USE_LABEL	GROUP_NO	GROUP_Letter	MalasseziaGenusLeve	Patient	Tissue	Malas	GENOME	TOTAL READS	TOTAL HUMAN	PERCENT READS ARE MALASSEZ
N_1	1	A	normal	1	Normal	yes	hg38	18858261	9085011	0.01[illegible]
T_1	2	B	moreinN	1	Tumor	yes	hg38	22821514	10788345	0.0[illegible]
N_2	1	A	normal	2	Normal	yes	hg38	34870246	16274139	0.17[illegible]
T_2	2	B	moreinN	2	Tumor	yes	hg38	28604529	13761918	0.02[illegible]
N_3	1	A	normal	3	Normal	yes	hg38	20067601	9752758	0.00[illegible]
T_3	2	B	moreinN	3	Tumor	yes	hg38	25180357	12250509	0.00[illegible]
N_4	1	A	normal	4	Normal	yes	hg38	17769109	8488343	0.00[illegible]
T_4	2	B	moreinT	4	Tumor	yes	hg38	39754255	19063222	0.01[illegible]
N_5	1	A	normal	5	Normal	yes	hg38	29585656	14189749	0.7[illegible]
T_5	2	B	moreinN	5	Tumor	yes	hg38	36737424	17795862	0.00[illegible]
N_6	1	A	normal	6	Normal	yes	hg38	34809441	16864905	0.01[illegible]
T_6	2	B	moreinT	6	Tumor	yes	hg38	24993439	12139287	0.02[illegible]
N_7	1	A	normal	7	Normal	yes	hg38	35439899	17087723	0.01[illegible]
T_7	2	B	moreinT	7	Tumor	yes	hg38	30130953	14606463	0.01[illegible]
N_9	1	A	normal	9	Normal	yes	hg38	40309681	19442161	0.031
T_9	2	B	moreinN	9	Tumor	yes	hg38	24666781	12105195	0.012
N_10	1	A	normal	10	Normal	yes	hg38	23369068	11433849	0.018
T_10	2	B	moreinT	10	Tumor	yes	hg38	33765469	16437724	0.021
N_11	1	A	normal	11	Normal	yes	hg38	14461851	6960809	0.046
T_11	2	B	moreinN	11	Tumor	yes	hg38	12230195	5977344	0.02
N_12	1	A	normal	12	Normal	yes	hg38	19113684	9313691	0.032
T_12	2	B	moreinT	12	Tumor	yes	hg38	65797247	31684330	0.054
N_13	1	A	normal	13	Normal	yes	hg38	22638160	11090895	0.102
T_13	2	B	moreinN	13	Tumor	yes	hg38	29198837	14100141	0.027
N_14	1	A	normal	14	Normal	yes	hg38	27131048	13192198	0.021
T_14	2	B	moreinT	14	Tumor	yes	hg38	42312723	20659150	0.034
N_15	1	A	normal	15	Normal	yes	hg38	38754908	18678438	0.009
T_15	2	B	moreinN	15	Tumor	yes	hg38	96452644	46639714	0.003
